# Efficacy of procalcitonin and pentraxin-3 as early biomarkers for differential diagnosis of pleural effusions

**DOI:** 10.1515/pp-2021-0111

**Published:** 2021-04-19

**Authors:** Anita Sharma, Apurva Agrawal, Girish Sindhwani, Ashish Sharma, Sojit Tomo, Jaykaran Charan, Dharmveer Yadav, Praveen Sharma

**Affiliations:** Himalaya Institute of Medical Science, Swami Rama Himalaya University, Dehradun, Uttrakhand, India; RNT Medical College, Rajasthan University of Health Science, Udaipur, Rajasthan, India; All India Institute of Medical Sciences, Rishikesh, Uttrakhand, India; Geetanjali Medical College, Geetanjali University, Udaipur, Rajasthan, India; All India Institute of Medical Sciences, Jodhpur, Rajasthan, India

**Keywords:** malignant effusions, parapneumonic effusions, pentraxin-3, pleural effusion, procalcitonin, tuberculous effusions

## Abstract

**Objectives:**

Pleural effusion, defined as an abnormal accumulation of fluid in pleural space, can be of two types: transudative and exudative. The primary aim of the study was to assess the predictive accuracy of procalcitonin (PCT) and pentraxin-3 (PTX-3) in comparison to other biochemical markers such as C-reactive protein (CRP), and adenosine deaminase (ADA) in the differential diagnosis of pleural effusions.

**Methods:**

A cross-sectional analytical study was conducted on patients with pleural effusion. Multiple comparisons and receiver-operating characteristics (ROC) analyses were made to evaluate the diagnostic significance of biochemical markers.

**Results:**

Sixty-six patients with exudative pleural effusion classified as malignant, tuberculous, and parapneumonic effusions (malignant pleural effusion [MPE], tuberculous [TPE], and parapneumonic [PPE]) were included. Significant differences in pleural fluid levels in both PCT (p-value: 0.001) and PTX-3(p-value: 0.001), as well as serum levels of PCT (p-value: 0.001), were observed between the three groups. ROC analysis showed both PTX-3 and PCT having favorable discrimination ability with high sensitivity (≥90%) and specificity to predict PPE from TPE and MPE.

**Conclusions:**

Evaluation of serum and pleural fluid PCT and levels of PTX-3 in the pleural fluid may be used as an early biomarker to differentiate the etiology of pleural effusion.

## Introduction

Pleural effusion is defined as an abnormal accumulation of fluid in pleural space [1]. It can be of two types: transudative and exudative. Diseases like cirrhosis of the liver, nephrotic syndrome, and congestive heart failure lead to transudative effusion, while infection and malignancy are the most common causes for exudative effusion [2]. Various modalities are available to diagnose parapneumonic (PPE), tuberculous (TPE), and malignant pleural effusion (MPE). These modalities include biochemical [3], microbiological [4], cytopathological analyses [5], and radiological techniques [6]. In addition to these modalities, there are at least two biomarkers showing promising results in the early diagnosis of pleural effusions: adenosine deaminase (ADA) and acute-phase proteins like C-reactive protein (CRP). These established biochemical markers (CRP and ADA) aid in the differential diagnosis of various etiologies’ pleural effusions. ADA is useful in diagnosing TPE [7], while acute-phase proteins like CRP aid in diagnosing PPE [8].

Recently, two further biomarkers have been evaluated in the early diagnosis of pleural effusions: pentraxin-3 (PTX-3) and procalcitonin (PCT). PTX-3 pleural fluid levels distinguish PPE and other exudative effusions [9], [10], [11], [12]. PTX-3, also known as tumor necrosis factor (stimulated gene 14), is a Pentraxin superfamily member. Although Pentraxin proteins are synthesized in the liver, they can also be produced locally in response to pro-inflammatory stimuli. PTX-3 is secreted by various cells like neutrophils, alveolar cells fibroblasts, adipocytes, and smooth muscle cells. PCT, a marker used in sepsis diagnosis, is also significant in discriminating PE of different etiologies [13], [14]. The lung and liver produce PCT after infections, especially bacterial infections [15], [16].

Thus, much research has been carried out in the last few years to validate biomarkers able to differentiate pleural effusions’ etiology. However, an adequate diagnostic accuracy of these biomarkers has not yet been demonstrated [17]. We planned a comparative analysis to assess the predictive accuracy and diagnostic significance of PCT and PTX-3 compared to established biochemical markers (CRP and ADA) in the early differential diagnosis of pleural effusions.

## Materials and methods

### Study population

Patients who underwent diagnostic or therapeutic thoracocentesis during the past year in the Department of Pulmonary Medicine, Himalaya Institute of Medical Science, Swami Rama Himalaya University, Dehradun, Uttrakhand, India, were enrolled in the study. Only patients with exudative pleural effusion were included in the study. Patients with pyothorax, hemothorax, or transudative effusion were excluded. Pleural fluid and serum samples from such patients were collected after obtaining informed written consent. Approval of the Institutional Review Board (IRB) was received before the study, and the IRB approval number was HIMS/RC/2017/40 dated 31/01/2017.

### Study design

This is an analytical cross-sectional study. All patients with exudative pleural effusion were divided into three groups after routine pleural fluid analysis based on widely accepted Light’s criteria [18].

– Group 1: Patients with TPE: 33 patients
  ○ Tuberculous effusions were confirmed by the presence of acid-fast bacilli (AFB) and TB-PCR testing.
– Group-2: Patients with MPE: 23 patients
  ○ Malignant effusions were confirmed by cytology/histology examination. All 23 MPE cases were cytologically confirmed and were further divided into primary and secondary.
– Group-3: Patients with PPE: 10 patients
  ○ PPE was confirmed by purulent sputum and response to antibiotics.

Samples were collected with stringent aseptic precaution from the Department of Pulmonary Medicine and send to the central diagnostic laboratory for evaluation.

### Laboratory analysis

Biochemical parameters, including protein, lactate dehydrogenase (LDH), CRP, and pentraxin, were performed immediately on automated analyzers. Serum/pleural fluid PCT sample was preserved at −20 °C before analysis as recommended by the kit insert. After routine cytological and microbiological examination, samples of serum and pleural fluid were analyzed for PTX3 (detection limit, 0.025 ng/mL), CRP (detection limit, 10 μg/mL), and PCT (detection limit, 27 pg/mL) using commercial kits on MR-96A ELISA reader and COBAS-6000 modular analyzer. ADA was estimated by the enzymatic method on Unicel DXC800 (Beckman Coulter India [Pvt.] Ltd). Assay kits were calibrated, and quality controls were analyzed daily at the National Accreditation Board for testing and calibration laboratories (NABL) accredited lab in Himalaya Institute of medical science. The diagnostic labs also participate in EQAS (monthly external quality assurance services) assurance programs to maintain the accuracy and precision of laboratory results.

### Statistical aspects

Data were analyzed by using statistical software SPSS 20. Categorical data were expressed as frequency and percentage. Quantitative data were expressed as mean ± SD and median (min–max) for normal and skewed distribution. Spearman correlation coefficient was used to find the correlation between biomarkers in serum and pleural fluid. Kruskal Wallis H test followed by Dunn’s posthoc multiple comparison tests to compare median values among the three groups. Receiver operating characteristics (ROC) curve was used to find cut-off values for pneumonia considering TB and lung cancer reference one by one. A ROC analysis was performed, and the area under the curve (AUC) was calculated.

## Results

### Patient’s baseline characteristics

Out of the total 80 patients enrolled, 14 were excluded after diagnosis of pyothorax, hemothorax, or transudative effusion. The remaining study group included 66 patients (52 males, 14 females) with a mean age of 53.9 years. Out of these patients, 33 (50%) had TPE, 23 (34%) had MPE, and 10 (15%) had PPE. Out of 23 MPE, 19 were due to primary lung cancer (14 adenocarcinoma, three small cell carcinoma, one squamous cell carcinoma, one large cell carcinoma). The remaining four effusions were secondary to other malignancies. The pleural fluid characteristics of the 66 patients are shown in Table 1.

**Table 1:** Baseline characteristics of patients with pleural effusion.

Variable		Tuberculous pleural effusion (TPE) n=33	Malignant pleural effusion (MPE) n=23	Parapneumonic pleural effusion (PPE) n=10
Age, years		47.6 ± 20.2	63.7 ± 13.6	52.4 ± 13.3
Sex	Male	25 (75.7%)	21 (91.3%)	6 (60.0%)
	Female	8 (24.2%)	2 (8.7%)	4 (40.0%)
Protein, g/dL	Pleural	3.85 ± 0.30	4.6 ± 0.20	3.72 ± 0.43
	Serum	6.29 ± 0.58	6.70 ± 0.55	6.06 ± 0.65
	PF/serum ratio	0.61	0.68	0.61
LDH, IU/L	Pleural	422.1 ± 117.4	507.2 ± 149.3	510.6 ± 171.8
	Serum	209.9 ± 22.09	272 ± 41.57	258.8 ± 41.59
	PF/serum ratio	1.91	1.83	1.98

TPE, tuberculous pleural effusion; MPE, malignant pleural effusion; PPE, parapneumonic pleural effusion.

### Biomarkers levels in the serum

Analysis of serum biomarkers revealed variations across the three groups (TPE, MPE, and PPE). However, only PCT showed a substantial difference between all three groups (TPE vs. MPE, TPE vs. PPE, and MPE vs. PPE) by multiple comparisons. ADA showed a significant difference only between TPE and PPE. Serum CRP and PTX-3 levels showed no statistical differences between the groups (Table 2).

**Table 2:** Biomarkers levels in serum according to etiology of pleural effusion.
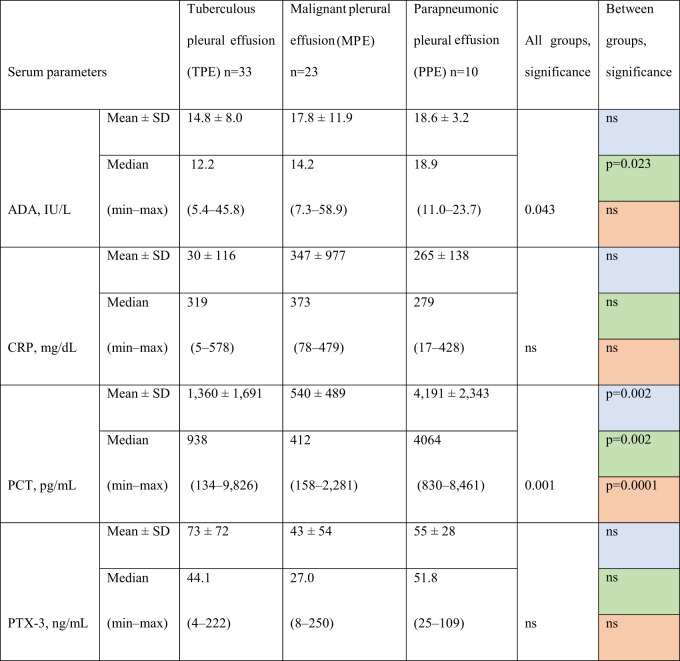

Comparisons. Blue fields: TPE vs. MPE. Green fields: TPE vs. PPE. Red fields: MPE vs. PPE. ADA, adenosine deaminase; PCT, procalcitonin; PTX-3, pentraxin-3; CRP, C-reactive protein; ns, not significant.

### Biomarkers levels in the pleural fluid

ADA, PCT, and PTX-3 levels in pleural fluid revealed notable differences across the groups. Only PCT showed a significant difference between all three groups (TPE vs. MPE, TPE vs. PPE, MPE vs. PPE) by multiple comparisons. ADA and PTX-3 levels were significantly different between two groups (TPE vs. MPE and MPE vs. PPE) and (TPE vs. PPE, MPE vs. PPE), respectively. CRP levels showed no statistically significant difference between the groups (Table 3).

**Table 3:** Biomarkers levels in the pleural fluid according to the cause of the effusion.
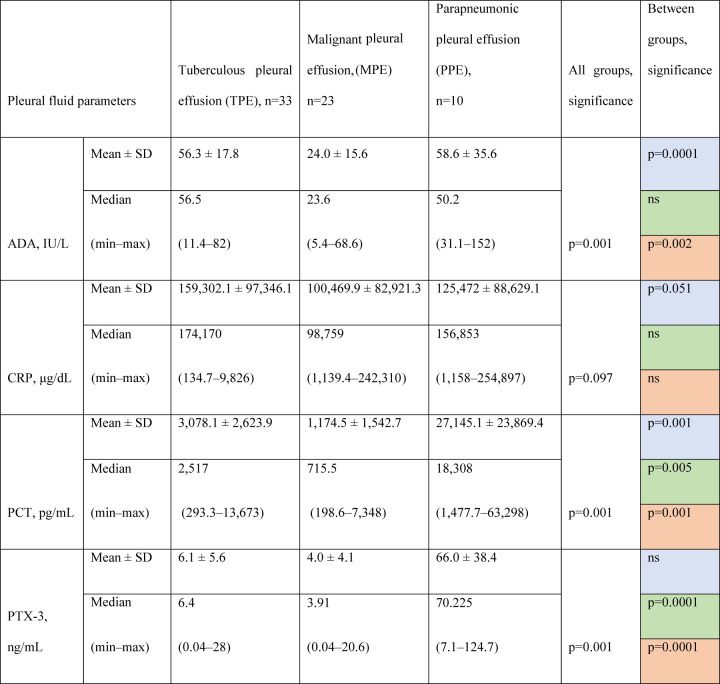

Comparisons. Blue fields: TPE vs. MPE. Green fields: TPE vs. PPE. Red fields: MPE vs. PPE; SD, standard deviation; ADA, adenosine deaminase; PCT, procalcitonin; PTX-3, pentraxin-3; CRP, C-reactive protein; ns, not significant.

### ROC analysis of the diagnostic performance of PCT and PTX3

**Figure 1:**
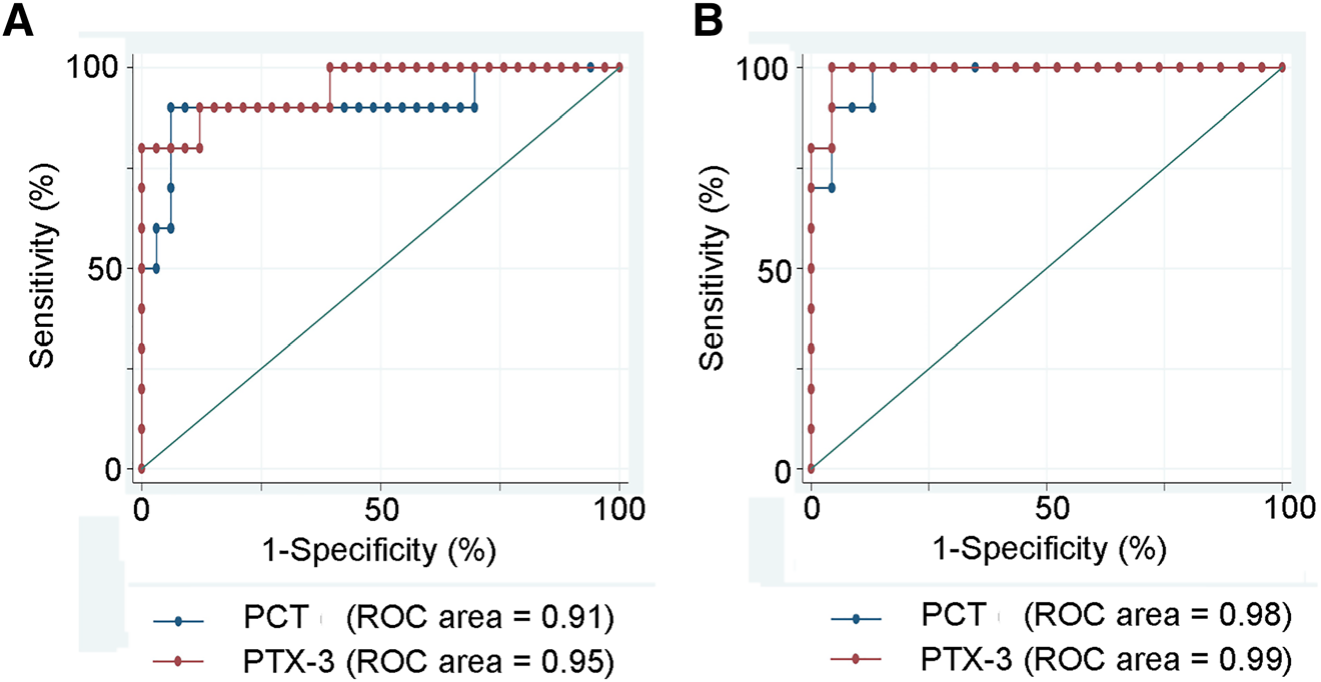
Prediction of a parapneumonic nature of pleural effusion. (A) Prediction of a parapneumonic nature of a pleural effusion (PPE) vs. a tuberculous nature (TPE). (B) Prediction of a parapneumonic effusion (PPE) vs. a malignant pleural effusion (MPE). The prediction, represented by the ROC area, was in both situations excellent (over 90%). The best prediction was reached using PTX-3 for PPE (ROC area = 99%). Areas under the curve (AUCs) produced by receiver operating characteristics.

**Figure 2:**
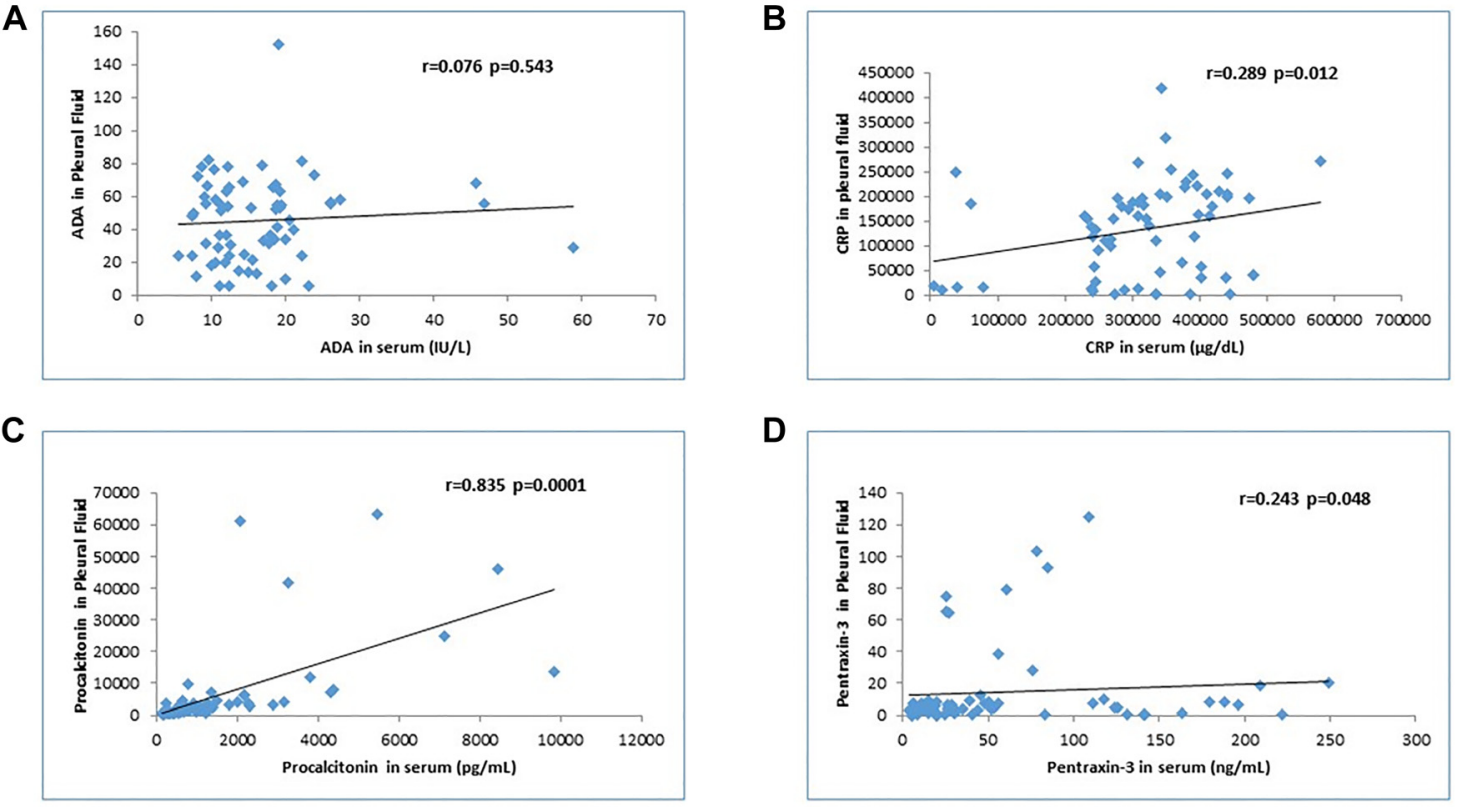
Analysis of the correlation between serum and pleural fluid levels of four biomarkers (ADA, CRP, PCT, and PTX-3). (A) Adenosine deaminase (ADA). (B) C-reactive protein (CRP). (C) Procalcitonin (PCT). (D) Pentraxin-3 (PTX-3). There is a significant correlation for CRP, PCT, and PTX-3 but not for ADA.

To differentiate PPE from TPE, pleural fluid PCT levels at the cut-off value of 6,173 had a sensitivity and specificity of 90%, and PTX-3 levels at the cut-off 9.2 had a sensitivity and specificity of 90 and 88%, respectively. To differentiate the PPE from MPE, pleural fluid PCT levels at the cut-off value of 2,189.7 had a sensitivity and specificity of 90 and 87%, respectively; PTX-3 levels at the cut off 7.19 had a sensitivity and specificity of 100 and 95.6%, respectively (Table 4, Figure 1). Correlation analysis showed a significant positive correlation for CRP, PCT, and PTX-3 in serum and pleural fluid (Figure 2).

**Table 4:** Predictive values of CT and PTX-3 for distinguishing a parapneumonic effusion (PPE) vs. malignant pleural effusion (MPE), respectively, a tuberculous pleural effusion (TPE).

	Biomarker	Cut-off value	AUC (95% CI)	Sensitivity	Specificity	LR+	LR−
To predict PPE from TPE	PCT, pg/mL	≤6,173	0.90 (0.77–1.00)	90	90	9.9	0.11
	PTX-3, ng/mL	≥9.2	0.94 (0.86–1.00)	90	88	7.4	0.11
To predict PPE from MPE	PCT, pg/mL	≤2,189.7	0.97 (0.94–1.00)	90	87	6.9	0.11
	PTX-3, ng/mL	≥7.19	0.99 (0.97–1.00)	100	95.6	23.01	0.000

AUC, area under the curve; LR+, likelihood ratio positive; LR−, likelihood ratio negative; PCT, procalcitonin, PTX-3, pentraxin-3; PPE, parapneumonic effusion; MPE, malignant pleural effusion; TPE, tuberculous pleural effusion.

## Discussion

The purpose of our study was to find unsurpassed abilities of biochemical markers in diagnosing and differentiating pleural effusion. We evaluated two established (ADA, CRP) and two more recent biomarkers (PTX-3 and PCT) in serum and pleural fluid separately to find their accuracy and efficiency to discriminate pleural effusion. Our study’s novelty was the analysis of different biomarkers simultaneously in the serum and pleural fluid, revealing that PTX-3 measurement in the pleural fluid is more accurate than in the serum. In contrast, both serum and pleural fluid levels of PCT provided a higher accuracy rate in discrimination of pleural effusions of different etiologies.

### Analysis of serum biomarkers for discriminating the origin of pleural effusions

The prior literature shows that PCT in serum and pleural fluid can differentiate between PPE and MPE [19]. Elevated serum PCT levels were also found in community-acquired pneumonia (CAP) vs. tuberculous patients [20]. The present comparative analysis of four biomarkers found that significantly elevated PCT in serum and pleural fluid differentiates between TPE, PPE, and MP in agreement with the previous studies.

### Analysis of pleural fluid biomarkers for discriminating the origin of pleural effusions

In the present study, biomarker analyses in the pleural fluid revealed that ADA, PCT, and PTX-3 in pleural fluid significantly differentiate PPE from TPE and MPE. ADA levels were found to be significantly higher in tuberculous exudative vs. nontuberculous exudative pleural effusions [21], [22], [23]. Similarly, PTX-3 in the pleural fluid could distinguish between PE of various etiologies and differentiate PPE from TPE and MPE. This result confirms previous data showing that pleural fluid levels of PTX-3 could significantly differentiate between PE of different etiologies [24], [25]. Estimating pleural fluid PCT levels in our study showed that PCT significantly distinguishes between PPE, TPE, and MPE. Again, this confirms previous studies showing that PCT levels can diagnose between PPE and non-PPE [25], [26]. Various studies suggested pleural fluid CRP is a better indicator to differentiate between PE of different etiologies [27], [28]. However, we found that pleural fluid CRP did not significantly distinguish between PPE, TPE, and MPE.

After determining the area under the curve of PCT and PTX-3 levels in the pleural fluid at a particular cut-off value, we found both biomarkers suitable to differentiate PPE from TPE. We set the cut-off value based on literature data. Wang et al. [26] reported that PCT levels could differentiate PPE from non-PPE at a cut-off point of 0.18 ng/mL with an AUC 0.776 (sensitivity, 69.7%; specificity, 72.1%). With the same cut-off point for PCT (0.18 ng/mL), Lin et al. [29] reported an AUC of 0.752 (sensitivity, 66.7%; specificity, 77.4%). Yeo et al. [30] found that PTX3 yielded the most constructive discriminating ability to predict PPE from MPE or TPE by providing the following: AUC, 0.74 (95% CI, 0.63–0.84), sensitivity, 62%; and specificity, 81% at a cut-off point of 25.00 ng/mL. Contrastingly, Porcel et al. [18] determined that PCT levels in the pleural fluid were of little value. Gabhale et al. [31] reported pleural fluid CRP to have good sensitivity (97.05%) and specificity (71.76%) in distinguishing TPE from non-TPE.

Our study also has certain limitations. Firstly, the sample size was relatively small, and there was a variation in cases accretion. Thus, our data has exploratory value, and the obtained cut-offs from ROC must be re-assessed in a large population to validate the findings. Secondly, sampling bias may have been present during the pleural fluid tapping procedure, which may have influenced the validity of laboratory measurements. We did not analyze the demographic and baseline patient characteristic (urban/rural, smoking, alcohol). Further laboratory data such as serum/pleural levels of glucose/HbA_1c_, albumin and serum/fluid cytology were not considered. A possible influence of the treatment applied (e.g., administration of chemotherapy, steroids, or antibiotics) on the laboratory results cannot be excluded.

There is no gold standard diagnostic technique for differential diagnosis of pleural effusion. The available traditional methods like cytology and microbiology are time-consuming. Depending on the laboratory consultant’s expertise, there is a chance of subjective bias affecting the patients’ management. Radiological investigations (USG/CT/MRI) are rapid but cannot distinguish the cause of the pleural effusion. Radiological studies are costlier, require specific technical infrastructure as well as diagnostic confirmation. The present study shows that newer biomarkers might be sensitive and specific enough compared to traditional methods. Although we did not evaluate these aspects, biomarker testing might be more cost-effective, rapid, reliable, and easy to perform than available comparators, improving the capacity to differentiate pleural effusion. Serum and pleural fluid PCT and PTX-3 levels might be useful early biomarkers to diagnose pleural effusions’ etiology. However, more extensive, adequately powered prospective studies are required to validate our findings.
